# HPLC/HRMS and GC/MS for Triacylglycerols Characterization of Tuna Fish Oils Obtained from Green Extraction

**DOI:** 10.3390/foods12061193

**Published:** 2023-03-11

**Authors:** Serena Indelicato, Vita Di Stefano, Giuseppe Avellone, Daniela Piazzese, Mirella Vazzana, Manuela Mauro, Vincenzo Arizza, David Bongiorno

**Affiliations:** 1 Department of Biological, Chemical and Pharmaceutical Science and Technology (STEBICEF), University of Palermo, Via Archirafi 32, 90123 Palermo, Italy; 2 Department of Earth and Marine Sciences (DISTEM), University of Palermo, Via Archirafi 22, 90123 Palermo, Italy

**Keywords:** tuna oil, triglycerides, fatty acids, FAMEs, PUFAs, mass spectrometry, accurate mass, omega-3 supplements

## Abstract

Background: Fish oil is one of the most common lipidic substances that is consumed as a dietary supplement. The high omega-3 fatty acid content in fish oil is responsible for its numerous health benefits. Fish species such as mackerel, herring, tuna, and salmon are particularly rich in these lipids, which contain two essential omega-3 fatty acids, known as eicosapentaenoic acid (EPA) and docosahexaenoic acid (DHA). Objectives: Due to the scarcity of information in the literature, this study aimed to conduct a qualitative and quantitative characterization of triglycerides (TAGs) in crude tuna fish oil using HPLC/HRMS. Fatty acid (FA) determination was also performed using GC/MS. The tuna fish oils analyzed were produced using a green, low-temperature process from the remnants of fish production, avoiding the use of any extraction solvents. Results: The analyses led to the tentative identification and semi-quantitation of 81 TAGs. In silico saponification and comparison with fatty acid methyl ester results helped to confirm the identified TAGs and their quantities. The study found that the produced oil is rich in EPA, DHA, and erucic acid, while the negligible isomerization of fatty acids to trans-derivatives was observed.

## 1. Introduction

Fish byproducts contain bioactive compounds such as proteins and n-3 polyunsaturated fatty acids (n-3 PUFAs), making them valuable resources that are often discarded as waste or used for animal feed or fertilizer [1]. However, these byproducts can be utilized to produce high-value-added products, reducing environmental pollution and increasing the competitiveness of the fishing industry [2]. The content of n-3 PUFAs in fish byproducts varies from 1.40% to 40.10%, depending on the species and tissue type [3]. Fish is considered the most common source of omega-3 in the human diet, with fatty fishes (such as the Salmonidae, Scombridae, and Clupeidae families) containing the highest percentages of long-chain fatty acids (FA), including eicosapentaenoic acid (EPA) and docosahexaenoic acid (DHA) [4].

Fish oil is widely recognized as a healthy product [5,6] due to its high levels of omega-3 PUFAs, which are more abundant than in microalgae or seed oils [4]. Fish oils are therefore ideal for producing concentrated omega-3 foods and supplements [5]. Scientific evidence suggests that consuming very long-chain ω-3 FAs, also known as “n-3 very long-chain polyunsaturated fatty acids” (n-3 VLC-PUFA), promotes the development of nervous systems and protects against some degenerative diseases [7]. In premature infants, DHA is associated with better cognitive and visual function [8,9]. Based on its beneficial effects, the American Heart Association recommends a daily intake of about 1 g of long-chain omega-3 FAs for people with known coronary heart disease. It has also been suggested that daily consumption of omega-3 FAs should not fall below 0.5 g, with the ideal intake being two to three times higher [6]. The pharmaceutical and food industries have shown increasing interest in the benefits of regular omega-3 FA consumption [5,6,8,10].

The synthesis of long-chain ω-3 fatty acids (n-3 LC-PUFAs) in humans occurs through the conversion of alpha-linolenic acid (ALA), which is present in significant amounts in vegetable oils and serves as their precursor. However, ALA is an essential fatty acid that cannot be produced by the body and must be obtained through the diet [11,12]. Although ALA can be easily obtained from vegetables, the conversion rate to n-3 LC-PUFAs is too low to meet physiological needs [13]. Therefore, vegetable oils alone cannot provide enough n-3 LC-PUFAs to guarantee healthy nutrition and reduce the risk of cardiovascular diseases [7]. This highlights the importance of including fish in the diet, as it is a valuable source of n-3 LC-PUFAs [14,15]. However, fish stocks are reducing [16,17], making it necessary to develop a long-term strategy for a renewable source of n-3 LC-PUFAs to meet future needs. One approach is the development of new oilseed crops containing n-3 LC-PUFAs. This vegetable source of n-3 LC-PUFAs can be grown, harvested, and used to produce functional foods. Although significant research has been done to produce such a crop [18,19,20,21,22,23,24], fatty fish still remain the primary source of this essential class of fatty acids [4]. Therefore, there is current interest in developing protocols to recover molecules of nutritional/nutraceutical interest, such as long-chain PUFAs, from agri-food and industrial production waste, creating a virtuous circular economy [25].

Fish oil is mainly composed of triglycerides (TAGs), constituting up to 95% of the oil content [26]. It can be produced through various methods, including hydraulic (cold) pressing [27], hexane extraction (for analytical purposes) [28,29], and supercritical fluid extraction (SFE) [4,30]. It is preferable to use less invasive recovery methods, as they should not introduce modifications to bioactive principles or hydrolysis of TAGs and reduce the presence of xenobiotic substances [31,32].

In the industry, crude fish oil undergoes several refining processes to separate phospholipids (degumming step) [33,34], eliminate free fatty acids, decrease oil acidity (using neutralization or de-acidification steps), remove smelly compounds, and absorb pigments or contaminants, such as heavy metals, dioxins, or PCBs [35,36]. Supercritical fluids extraction (SFE) has been evaluated as a way to reduce thermal and chemical degradation during crude fish oil production, but its main limitation is the high cost at the production scale. Another approach to fish oil production is cold pressing, which leads to minor or negligible degradation of the PUFAs [37]. Cold presses consist of screw devices that extract fat (oil) from the fish paste. Although cold pressing usually leads to lower yields, it also produces higher quality oils [24]. The paste is obtained by first wet reducing the tissues, achieved by cooking the crushed raw material at 85/95 °C [31]. After cold pressing, the paste is centrifuged to separate the oil from water and solid residues. This simple and green process can be performed continuously and allows for obtaining high volumes of crude fish oil.

Another important industrial process is fish oil deodorization, aimed at improving its sensory appeal and extending its use in the food and cosmetic industry. This step is commonly based on the application of elevated temperatures [31], but treatments above 180 °C imply significant PUFA degradation, involving chemical polymerization, isomerization, and TAGs hydrolysis [33]. The thermal degradation that occurs is almost exclusively of geometrical nature and leads to more stable trans isomers, leaving the double bonds in the same position [38]. However, the derivatives produced are often of negligible biological activity and, in the worst cases, dangerous for human health [39]. Therefore, some approaches have been developed to reduce thermal degradation, such as oil adsorption on resinous materials [40], vacuum steam distillation at low temperatures followed by silica gel column purification [40], or the adoption of diatomaceous earth [33].

Preserving the beneficial biological activity of TAGs, which constitute the vast majority of fish oils, is therefore paramount. The esterification of fatty acids with glycerol backbone to give TAGs has a profound influence on the intestinal absorption of DHA and EPA [41]. Therefore, the quantitative TAGs composition of fish oil is not trivial information, yet it is lacking in the literature.

In this study, we performed the qualitative and quantitative characterization of the TAGs and fatty acid composition of tuna fish oil, as yellowfin tuna (*Thunnus albacares*) heads and remnants are rich in n-3 PUFAs, especially DHA [1,42,43,44]. The crude tuna oil was obtained by a mild production method involving tuna fish remnant mincing, mild heating of the paste, and oil separation by centrifugation. This green and solvent-free procedure allowed for obtaining a sustainable production. Since mass spectrometry is one of the most valuable and reliable tools for determining analytes of vastly different natures [45,46], we used liquid chromatography high-resolution mass spectrometry (LC-HRMS) for triglyceride recognition and semi-quantitation, while a GC/MS apparatus was used for fatty acid methyl ester determination.

## 2. Materials and Methods

Four samples of tuna oil were produced independently, according with the procedure described by Yinet al. [47] and by Inguglia et al. [48] with slight variations. For the production, heads and other tuna wastes consisting of skin, fishbones, offal, and gullets (1 kg each) belonging to different lots of mixed raw material were used. The offal did not comprise liver, spleen, and pancreas. The remnants were minced with an IKA MF 10 meat grinder and subsequently homogenized using an Ultraturrax IKA T25 digital high-speed homogenizer with S 25 NK—19 G dispersion probe. The homogenate was subsequently diluted with distilled water at a ratio of 1: 1. The oil extraction was performed at 50 °C for 60 min. After heat treatment, the sample was filtered to obtain the liquid fraction and subsequently centrifuged at 10,000× *g* at 4 °C in a variable angle rotor (Beckman J6-M) centrifuge to separate the aqueous phase from the oily component. The centrifugation of the homogenate led to an oily phase that was well-distinguished at the top, followed, in an intermediate position, by the aqueous phase, and finally, at the bottom of the test tube, by the insoluble component that had passed through filtration. The tuna oil, layered at the top of falcon tubes, was very carefully recovered by a Pasteur pipette. The fish oil extraction process was conducted in the test tubes. After the homogenate was transferred into the tube and capped tightly, oil extraction was performed as described previously. The sample preparation of FAMEs for the analytical determination by GC/MS was carried out in accordance with the International Olive Council [49] protocol. The procedure for FAMEs determination foresees the dissolution of approximately 0.1 g of oil in a 5 mL screw top test tube with 0.2 mL KOH (2 N) in methanol solution and 1 mL n-hexane. The solution was vigorously shaken for 30 min and left to stratify until the upper solution became clear.

For GC analysis, a Thermo Fisher ISQ was used, equipped with a Trace 1300 GC. The chromatographic column was a Supelcowax (30 m × 0.32 mm, 0.25 µm film thickness) with the following temperature program: starting temperature 120 °C (5 min hold), temperature gradient at 15 °C/min up to 200 °C, temperature gradient at 10 °C up to 250 °C (8 min hold). The split/splitless injector was set at 270 °C in splitless mode for 1 min. The injection volume was 1 µL. The ISQ mass spectrometer was operated in full scan (50–400 *m*/*z*), positive ion mode, with the EI source at 270 °C and an electron ionization potential of 70 V. The transfer line between GC and MS was set at 260 °C.

Sample preparation for the analytical determination of TAGs by LC/HRMS was achieved by dissolving 0.5 μL of oil in 1.5 mL of methanol. The samples thus prepared were placed in an ultrasonic bath for 5 min to facilitate the dissolution.

LC-APCI/MS TAGs determination was conducted on a Waters Q-Tof Premier coupled with an Alliance 2695 (Waters) HPLC system equipped with an autosampler, degasser, and column heater. The compounds were chromatographically separated by a Thermo Hypersyl Gold column (5 cm × 2.1 mm i.d., particle size 1.8 µm) under the following conditions: column temperature, 25 °C; injected volume, 5 µL. The chromatographic details are reported in [50]. All samples were injected in duplicate. The MS experiments were performed using dynamic range enhancement (DRE) acquisition mode, which avoids MCP saturation, keeping a good sensitivity. This allows for correctly quantifying very abundant as well as trace-level compounds. Atmospheric pressure chemical ionization (APCI) was used in positive ion mode under the following conditions: corona probe current, 4 µA; corona voltage, 3.6 kV; probe temperature, 450.0 °C; sampling cone, 19.0 V; extraction cone, 4.3 V; ion guide, 1.2 V; source temperature 90 °C, cone gas, N_2_, flow of 50.0 L/h; desolvation gas, N_2_, flow of 600.0 L/h.

## 3. Results and Discussion

The analysis of triacylglycerols (TAGs) in tuna fish oil is not a new topic, but the available data in the literature are few and of a qualitative nature. Furthermore, quantitative data on TAGs are still scarce, and the reported composition of tuna fish oil varies depending on the tissues used for crude oil production [51,52,53]. Therefore, gathering further data to establish a reliable baseline is advisable.

Our work aimed to fill these gaps through a parallel determination of TAGs and FAMEs. The findings of the two analyses were compared quantitatively in a synergistic approach. It is, indeed, possible to obtain FAMEs levels in oils from the computational re-elaborations of the relative abundances of TAGs [54,55] determined by HPLC/HRMS using electrospray or, more frequently, APCI sources. The base principle is that the quantitative and qualitative pieces of information of FAMEs are both present in the relative abundances of the triglyceride components [50,56].

Using in silico saponification (ISS), it was possible to determine the fatty acid (FA) composition of an oily matrix from TAGs data. The identified TAGs and their corresponding abundances, organized into a datasheet, could be processed using free software called TAGSCHECK. The algorithm applied in TAGSCHECK is based on two assumptions: (a) the FA composition of the oils is derived almost exclusively from the TAGs, and (b) the identifications and relative abundances of the TAGs are reliable. Therefore, from an experimental point of view, one-third of the area of the TAG analyzed is allocated to each FA constituting the triglycerides. The total area of each fatty acid is due to the sum of its corresponding area fractions, and the FA results are expressed as a relative weight/weight percentage. In this approach, the quantitative determination of TAGs requires consideration of the differing ionization efficiencies that characterize each TAG. While reliable studies are not present for electrospray ionization, probably due to some drawbacks of this ionization type [57,58], for most of the TAGs (but not for all), relative ionization efficiencies (for APCI-generated ions) are reported in the literature [59,60] and have been implemented in the latest version of the free TAGSCHECK software. For TAGs with unknown relative ionization efficiency, the same ionization efficiency of triolein (OOO) has been introduced in the software. Good correlations between FAMEs, determined by GC/MS, and those obtained by ISS have been previously reported [50,61]. By reverse reasoning, TAGs determination could be considered reliable if the FAMEs levels determined by ISS are equal or very close to those obtained from GC/MS.

**Figure 1:**
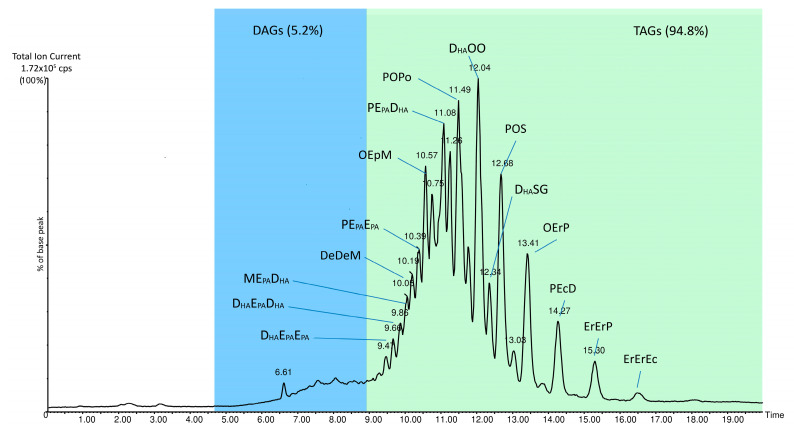
Total ion current HPLC/MS trace of the tuna fish oil. L = linoleic acid, P = palmitic acid, Po = palmitoleic acid, O = oleic acid, S = stearic acid, Ec = eicosaenoic acid, E_PA_ = eicosapentaenoic acid, M = myristic acid, D_HA_ = docosahexaenoic acid; Er = erucic acid.

**Figure 2:**
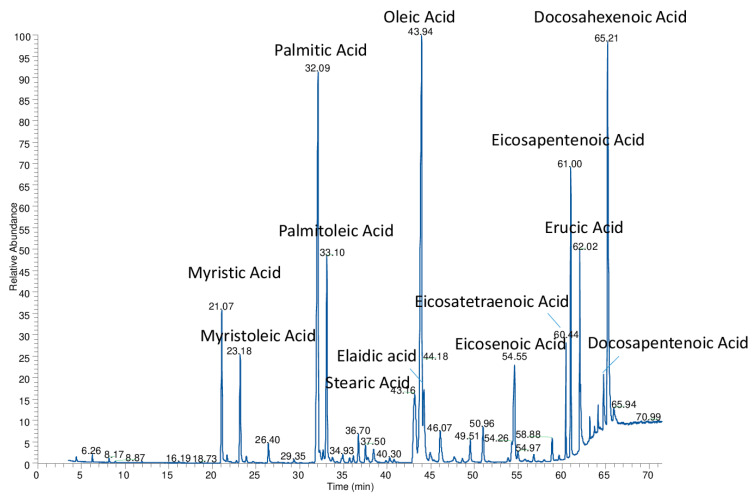
GC/MS chromatogram of fatty acid methyl esters (FAMEs) obtained from tuna fish oil.

The analysis of FAMEs through ISS has a noteworthy advantage, as it can be applied retrospectively to existing data in the literature. In some cases, it is even possible to improve the reliability of TAGs attribution a posteriori [50]. It is important to remember that TAGs are numerous, and many of them are isomers with identical molecular weights, making correct identification complicated. To differentiate them, it is necessary to determine the mono- and diglyceride fragments produced or perform a fine HRMS/MS^n^ analysis [61,62]. However, the latter approach, which is undoubtedly the best, requires appropriate equipment and high competency. A preliminary analysis with TAGSCHECK of the results obtained by Zhang et al. [63]—the only author reporting quantitative results of TAGs in tuna fish oil—showed that their quantitative analysis of the TAGs may not be accurate enough. From these data, the percentage of DHA, oleic acid (O), and EPA overlapped with their FAMEs percentage determined by GC. The levels of other fatty acids, even abundant ones, differed significantly from the quantities determined with TAGSCHECK based on these quantitative data of the TAGs. Zhang et al. [63] did not report the levels of erucic acid, which other authors have reported to be present in fair amounts, ranging from 2 to 10% in tuna oils [64]. Conversely, in Zhang’s study [63], DHA was much higher than that found by us and Truzzi et al. [65], and the percentages (justifiable based on the TAGs composition) were low for stearic acid and high for palmitic acid. It is important to note that Zhang’s approach is not based on the physical separation of TAGs by chromatography, which is by far the preferred approach today [60,62]. Our TAGs separation results achieved by LC/MS are shown in Figure 1. The diacylglyceridic fraction eluted at a lower retention time and constituted only the 5.2% of the total area of the chromatographic trace, recorded as total ion current. Considering their small abundance and according to previous studies [50], we did not take into account diacylglycerols (DAGs) for FA determination by ISS. FAMEs obtained from tuna oil transesterification are shown in Figure 2. Our data show that good amounts of DHA and EPA were found, along with a fair amount of erucic acid. These three high-molecular-weight acids made up over one-third of the entire oil composition. It is also worth noting that the percentage of trans oleic acid in all the crude oils analyzed was low, between 1–2% of the total FA content.

The chemical characterization of the extracted oil was performed by determining FA methyl esters analyzed by GC/MS and triglycerides (TAGs) by an HPLC/HRMS approach based on a ballistic gradient. The relative TAGs abundances, obtained from APCI/MS, also constituted the ISS data input. As aforesaid, for tuna fish oil, it has not always been possible to determine the APCI relative ionization efficiencies of all the TAGs (from the literature) nor to obtain the corresponding TAGs standard to experimentally determine it. Even taking into consideration this limitation that leads to some discrepancies for the lowest abundant FA, a fairly good agreement between fatty acid composition determined from FAMEs and from ISS was observed (Table 1).

Our FA results were also compared with data in the literature [59] based on commercial tuna fish oil and were found to be similar. In contrast, our study identified 81 TAGs (Table 2), which is several more than in other studies [63]. Triglycerides in Table 2 were tentatively identified based on the accurate masses (AM) of quasi-molecular and corresponding diglyceride fragment ions. This approach cannot definitively characterize regioisomers, as it would require more complicated MS^n^ analyses that are not possible with our current instrumental configuration. Nevertheless, the triglyceride attribution based on quasi-molecular ion and DAGs fragment accurate masses is reliable enough and only leaves the acidic residue position in the glycerol backbone undetermined (nominal).

Table 3 reports the quantitative results of TAG determination. The mass abundance of each triglyceride ranges from a maximum value of about 10% to a minimum value of 0.04%. In all cases, a distinct peak of the corresponding extracted ion signals was found with respect to the baseline. It is worth noting that the adoption of a ballistic chromatographic elution method led to a fast chromatographic run, which shows the elution of diglyceridic compounds (4–7 min) at lower retention time. However, the fast chromatography tradeoff is the co-elution of several substances in a short time, which leads to an increase in the total ion current and a perceived (but not real) raising of the chromatographic baseline. Despite this, the column resolution, as well as the extracted ion baseline, is still good and can be easily evidenced by exploiting the selectivity of the high-resolution mass analyzer.

Table 3 shows how 50% of the total mass of TAGs consists of the first 15 most abundant triglycerides, which represent less than 20% of the total number of TAGs detected. From the relative abundances of this reduced fraction of TAGs, it would be still possible to derive an indicative composition of the corresponding fatty acids (not shown).

It is worth noting that the relative percentage variation (RSD%) of triglyceride content in samples from the same oil was generally below 20% RSD (not shown). However, our findings revealed a significantly higher variation in triglyceride content when different oils, obtained from distinct remnants, were considered (Table 3). This once again highlights how the triglyceride composition of tuna oil is strongly influenced by the waste material used.

Our low-temperature green extraction process, which takes place at temperatures well below 180 °C, is simple, quick, and requires only small amounts of raw materials. Moreover, it avoids the isomerization reactions of the PUFA, as indicated by the absence of relevant amounts of isomerized derivatives in the FAMEs GC chromatograms at different retention times.

Nevertheless, our tuna oil still retains a slightly fishy flavor, which limits its potential use in cosmetics or human consumption products. However, a soft deodorization process could address this issue without compromising the overall quality of the high-value nutrients contained therein [66,67]. It is worth noting that the oil produced has very low humidity content, making it suitable for supercritical fluid extraction procedures and avoiding the need for an expensive freeze-drying process.

## 4. Conclusions

Tuna oil composition reported in the literature is markedly varied, from both qualitative and quantitative points of view, which our data confirm. The composition is mostly influenced by the tissues used for oil production (muscles, scales, bones, heads, stomach), but the extraction procedures have a non-negligible influence. It is also difficult to verify if any of the differences observed in the oil composition can be ascribed to a high-temperature processing step introduced to deodorize the matrix and eliminate the fishy smell of this fat material.

To date, only a few articles have dealt with the triglyceride (TAG) composition of tuna oil, and only one reports quantitative data. This study aimed to fill some gaps in this respect and successfully identified and semi-quantitatively determined 81 different TAGs. Among these, the four most abundant (D_HA_OO, OE_PA_M, D_HA_PO, E_PA_OP) contained EPA or DHA. The individual fatty acid compositions of the TAGs, determined by the identified TAGs and their relative abundance, agree with the fatty acids determined by the traditional FAMEs protocol, suggesting accurate identification and quantification of the TAGs. Finally, the negligible amount of trans-isomerized fatty acids suggests that our mild approach, mainly based on remnant mincing and centrifugation, could be considered as a good approach to obtain a quality raw fish oil.

## Figures and Tables

**Table 1:** Fatty acid composition of tuna fish oil determined by independent approaches.

Fatty Acids *	M	Po	P	Ml	Ln	L	O + El	S	Ec	E_PA_	Er	D_HA_	Dp	Et
	C14:0	C16:1	C16:0	C14:1	C18:3	C18:2	C18:1	C18:0	C20:1	C18:5	C22:1	C22:6	C22:5	C20:4
ISS (%)	5.2	6.6	20.2	0.8	0.8	1.2	22.8	4.3	1.5	11.4	8.3	12.1	2.4	2.2
FAMEs (GC/MS) (%)	5.7	7.6	18.1	0.6	1.2	1.5	18.3	4.2	3.1	11.1	11.4	12.4	1.3	3.5
Rel. Variation (%)	−9	−13	+12	+44	−38	−20	+24	+2	−52	+3	−27	−2	+88	−36

M = myristic acid, Po = palmitoleic acid, P = palmitic acid, Ml = myristoleic acid, Ln = linolenic acid, L = linoleic acid, O = oleic acid, El = elaidic acid S = stearic acid, Ec = eicosenoic acid, E_PA_ = eicosapentaenoic acid, Er = erucic acid, D_HA_ = docosahexaenoic acid, Et = eicosatetraenoic acid, Dp = docosapentaenoic acid. (*) Only FA with abundance > 0.5% have been reported.

**Table 2:** Triglycerides tentatively identified based on the accurate masses (AM) of quasi molecular and diglyceride protonated fragment ions. The acids position is only nominal (not determined by MS/MS experiments).

TAGs *	TAG^+^ AM	^#^ DAG1^+^ AM	DAG2^+^ AM	DAG3^+^ AM	TAGs	TAG^+^ AM	DAG1^+^ AM	DAG2^+^ AM	DAG3^+^ AM
EcErD_HA_	1015.86	687.61	677.53	1015.86	OErP	915.83	659.59	577.51	915.83
D_HA_LgS	1019.89	735.62	651.52	1019.89	SPEr	917.84	579.53	661.60	917.84
D_HA_D_HA_D_HA_	1023.73	695.49	695.49	1023.73	PoE_PA_D_HA_	923.70	595.46	621.48	923.70
D_HA_D_HA_Dp	1025.75	695.49	697.50	1025.75	D_HA_D_HA_M	923.70	695.49	595.46	923.70
ErErEc	1025.93	715.64	687.61	1025.93	D_HA_StO	925.54	643.28	649.51	925.54
MME_PA_	797.66	495.43	569.44	797.66	PE_PA_D_HA_	925.72	597.48	623.49	925.72
PPoP	805.72	549.48	551.49	805.72	PDpE_PA_	927.73	625.51	597.48	927.73
MOP	805.72	549.48	523.46	805.72	PEtD_HA_	927.73	599.49	623.49	927.73
PoPoO	829.72	547.46	575.49	829.72	D_HA_SO	933.78	651.52	649.51	933.78
OOM	831.74	603.53	549.48	831.74	PEcD	943.86	605.54	633.57	943.86
PPO	833.75	551.49	577.51	833.75	DpOS	935.80	651.52	653.54	935.80
LnMlE_PA_	845.65	543.43	619.46	845.65	D_HA_SS	935.80	651.52	651.52	935.80
OE_PA_M	851.70	623.49	549.48	851.70	ErOO	941.84	659.59	659.59	941.84
ErDpP	963.83	707.58	633.57	963.83	MErEr	943.86	605.54	605.54	943.86
PPE_PA_	853.72	551.49	597.48	853.72	D_HA_D_HA_Po	949.72	695.49	621.48	949.72
PPEt	855.73	551.49	599.49	855.73	EcEeE_PA_	951.73	649.50	651.52	951.73
MlEcO	857.75	575.49	547.46	857.75	OE_PA_D_HA_	951.73	623.49	649.51	951.73
POPo	831.74	577.51	549.48	831.74	D_HA_D_HA_P	951.73	695.49	623.49	951.73
D_HA_OO	931.77	649.51	649.51	931.77	PDpD_HA_	953.75	625.51	623.49	953.75
POS	861.79	577.51	579.53	861.79	DpE_PA_S	955.76	671.49	653.54	955.76
PoLnE_PA_	873.69	571.46	595.46	873.69	D_HA_SG	961.81	651.52	677.54	961.81
EcMlE_PA_	877.71	575.49	651.52	877.71	OErE_PA_	961.81	659.59	623.49	961.81
LnOLn	877.72	599.49	595.46	877.72	SErE_PA_	963.83	661.60	625.51	963.83
D_HA_PPo	877.72	623.49	621.48	877.72	D_HA_E_PA_E_PA_	971.70	669.47	669.47	971.70
E_PA_OP	879.73	623.49	597.48	879.73	ErErP	971.89	715.64	633.57	971.89
D_HA_PP	879.73	623.49	623.49	879.73	D_HA_D_HA_O	977.75	695.49	649.51	977.75
POEt	881.75	577.51	599.49	881.75	SErD_HA_	989.84	661.60	651.52	989.84
OLO	883.77	601.51	603.53	883.77	D_HA_E_PA_D_HA_	997.71	669.47	695.49	997.71
PoPEr	887.80	549.48	631.55	887.80	D_HA_DpE_PA_	999.73	697.50	669.47	999.73
E_PA_E_PA_Po	897.68	643.46	595.46	897.68	D_HA_EtD_HA_	999.73	671.49	695.49	999.73
ME_PA_D_HA_	897.68	569.44	595.46	897.68	ErErS	999.92	715.64	661.60	999.92
PE_PA_E_PA_	899.70	597.48	597.48	899.70	ME_PA_E_PA_	871.67	569.44	569.44	871.67
MaEcO	901.81	619.55	591.53	901.81	PoErEr	969.87	631.55	631.55	969.87
OLE_PA_	903.73	601.51	623.49	903.73	ErErEr	1053.96	715.64	715.64	1053.96
E_PA_OO	905.75	623.49	623.49	905.75	PoPoEr	885.78	547.46	631.55	885.78
D_HA_PO	905.75	623.49	649.51	905.75	ErErO	997.90	715.64	659.59	997.90
DpOP	907.77	651.52	625.51	907.77	D_HA_ErEr	1043.88	705.57	705.57	1043.88
D_HA_SP	907.77	651.52	623.49	907.77	D_HA_D_HA_Er	1033.81	695.49	705.57	1033.81
EtOS	909.78	625.51	627.52	909.78	D_HA_MEr	933.78	595.46	705.57	933.78
PoLEr	911.80	573.48	631.55	911.80	D_HA_ErEc	1015.85	705.57	677.53	1015.85
PoErO	913.81	631.55	575.49	913.81					

* Acidic residues: D = docosenoic, D_HA_ = docosaexaenoic, Dp = docosapentaenoic, Ec = eicosaenoic, E_PA_ = eicosapentaenoic, Er = erucic, Et = eicosatetraenoic, G = gadoleic, L = linoleic, Lg = lignoceric, Ln = linolenic, M = miristic, Ma = margaric, Ml = miristoleic, O = oleic, P = palmitic, Po = pamitoleic, S = stearic, St = C18:4. ^#^ For a protonated TAG containing the ABC acidic residues linked to the glycerol backbone, the fragments scheme is the following: DAG1^+^ = (TAG-C)^+^; DAG2 = (TAG-B)^+^; DAG3 = (TAG-A)^+^.

**Table 3:** Triglycerides tentatively identified and their relative abundances in *w*/*w* percent.

TAG	Tuna-1	Tuna-2	Tuna-3	Tuna-4	Average		%RSD
EcErD_HA_	0.50	0.14	0.32	0.31	0.32	±	47.3
D_HA_D_HA_D_HA_	0.04	0.02	0.03	0.03	0.03	±	20.7
D_HA_D_HA_Dp	0.02	0.01	0.02	0.02	0.02	±	27.4
ErErEc	0.13	0.00	0.07	0.07	0.07	±	80.7
MME_PA_	0.34	0.50	0.43	0.42	0.42	±	15.5
PPoP	0.41	0.62	0.51	0.52	0.52	±	16.9
MOP	6.56	9.65	8.38	7.98	8.14	±	15.6
PoPoO	1.43	2.03	1.73	1.70	1.72	±	14.2
OOM	1.05	1.48	1.31	1.21	1.26	±	14.3
PPO	3.43	4.22	3.76	3.81	3.80	±	8.5
LnMlE_PA_	0.76	0.94	0.86	0.87	0.86	±	8.4
OE_PA_M	4.21	5.01	4.36	4.77	4.59	±	8.0
DDpP	2.61	2.58	2.66	2.46	2.58	±	3.3
PPE_PA_	1.88	2.58	2.18	2.28	2.23	±	13.0
PPEt	1.93	2.06	1.98	1.94	1.98	±	2.9
MlEcO	1.19	1.56	1.40	1.43	1.40	±	11.0
POPo	2.38	2.81	2.57	2.63	2.60	±	6.8
D_HA_OO	3.71	4.56	4.10	4.20	4.14	±	8.4
POS	1.39	1.44	1.48	1.45	1.44	±	2.4
PoLnE_PA_	0.08	0.12	0.10	0.10	0.10	±	15.3
EcMlE_PA_	0.87	0.47	0.63	0.80	0.69	±	26.5
LnOLn	0.53	0.74	0.60	0.66	0.63	±	14.1
D_HA_PPo	2.47	3.43	2.81	3.06	2.94	±	13.8
E_PA_OP	4.10	3.72	3.42	4.39	3.91	±	10.9
D_HA_PP	1.29	1.77	1.74	1.36	1.54	±	16.2
POEt	2.22	2.48	2.58	2.32	2.40	±	7.0
OLO	1.46	1.12	1.36	1.30	1.31	±	10.8
PoPEr	3.41	3.47	3.69	3.38	3.49	±	4.9
E_PA_E_PA_Po	0.87	0.90	0.91	0.88	0.89	±	3.9
ME_PA_D_HA_	0.42	0.58	0.52	0.48	0.50	±	13.5
PE_PA_E_PA_	1.67	2.06	2.00	1.88	1.90	±	8.7
MaEcO	0.31	0.07	0.22	0.16	0.19	±	51.3
OLE_PA_	1.94	2.47	2.29	2.19	2.22	±	9.5
E_PA_OO	1.83	2.02	1.92	1.99	1.94	±	5.0
D_HA_PO	3.53	4.43	4.08	3.84	3.97	±	9.5
DpOP	1.54	1.59	1.65	1.54	1.58	±	3.6
D_HA_SP	2.58	2.45	2.75	2.26	2.51	±	9.4
EtOS	1.92	1.09	1.62	1.46	1.52	±	21.6
PoLEr	0.77	0.54	0.87	0.86	0.76	±	19.2
PoErO	1.73	1.21	1.84	1.86	1.66	±	17.3
OErP	2.86	2.03	4.61	4.47	3.49	±	33.3
SPEr	0.28	0.49	0.40	0.37	0.38	±	20.8
PoE_PA_D_HA_	0.71	0.24	0.50	0.47	0.48	±	36.7
D_HA_D_HA_M	0.20	0.26	0.22	0.23	0.23	±	12.5
D_HA_StO	1.23	1.48	1.40	1.35	1.37	±	7.1
PE_PA_D_HA_	1.41	1.70	1.59	1.53	1.56	±	8.0
PDpE_PA_	0.93	1.07	1.09	0.95	1.01	±	8.7
PEtD_HA_	0.47	0.46	0.48	0.44	0.46	±	4.5
D_HA_SO	1.49	1.08	1.41	1.16	1.29	±	16.3
PEcDe	0.00	0.01	0.01	0.01	0.01	±	56.0
DpOS	0.29	0.23	0.28	0.24	0.26	±	12.8
D_HA_SS	1.84	1.51	1.73	1.60	1.67	±	8.4
ErOO	1.15	0.57	1.27	1.26	1.04	±	45.5
MErEr	1.77	0.67	2.30	2.22	1.74	±	40.0
D_HA_D_HA_Po	0.44	0.48	0.43	0.50	0.46	±	7.9
EcEeE_PA_	1.22	1.23	1.31	1.15	1.23	±	6.6
OE_PA_D_HA_	1.22	0.86	1.12	1.00	1.05	±	14.8
D_HA_D_HA_P	0.78	0.86	0.93	0.76	0.83	±	11.1
PDpD_HA_	1.20	0.41	0.84	0.78	0.81	±	37.7
DpE_PA_S	1.20	0.41	0.86	0.80	0.82	±	37.2
D_HA_SG	1.95	1.29	1.72	1.57	1.63	±	15.9
OErE_PA_	0.75	0.38	0.76	0.79	0.67	±	27.2
SErE_PA_	0.45	0.35	0.81	0.81	0.60	±	37.0
D_HA_E_PA_E_PA_	0.28	0.22	0.26	0.24	0.25	±	8.9
ErErP	0.92	0.23	1.14	1.17	0.87	±	46.8
D_HA_D_HA_O	0.69	0.68	0.70	0.65	0.68	±	3.8
SErD_HA_	0.57	0.10	0.63	0.64	0.48	±	49.3
D_HA_E_PA_D_HA_	0.09	0.07	0.12	0.03	0.08	±	73.4
D_HA_DpE_PA_	0.09	0.04	0.06	0.06	0.06	±	31.6
D_HA_EtD_HA_	0.05	0.13	0.10	0.08	0.09	±	35.1
ErErS	0.17	0.00	0.18	0.19	0.13	±	61.4
ME_PA_E_PA_	0.82	0.86	0.87	0.80	0.84	±	6.9
ErErEr	0.06	0.00	0.06	0.06	0.04	±	61.9
PoPoEr	2.29	2.30	2.58	2.56	2.43	±	6.4
ErErO	0.37	0.09	0.44	0.43	0.33	±	45.8
D_HA_ErEr	0.22	0.03	0.22	0.23	0.18	±	51.8
D_HA_D_HA_Er	0.72	0.05	0.79	0.79	0.58	±	56.9
D_HA_MEr	0.35	0.31	0.39	0.39	0.36	±	10.6
D_HA_ErEc	0.51	0.15	0.50	0.59	0.44	±	42.7

## Data Availability

Data is contained within the article.
